# Effects of Various Flavors of Baijiu on the Microbial Communities, Metabolic Pathways, and Flavor Structures of Dongbei Suancai

**DOI:** 10.3390/foods13132015

**Published:** 2024-06-26

**Authors:** Xiao Li, Xingzhuang Wu, Yanqiu Han, Chen Wang, Lifeng Li, Xiaoli Zhang

**Affiliations:** Institute of Food and Processing, Liaoning Academy of Agricultural Sciences, Shenyang 110161, China; wuxingzhuang@126.com (X.W.); hyqiu2017@126.com (Y.H.); wangchen0913@163.com (C.W.); lifeng.li@sohu.com (L.L.); zxli2022@163.com (X.Z.)

**Keywords:** Baijiu flavor, Dongbei Suancai, microbial community, differential metabolic pathways, flavor structure

## Abstract

This study aimed to assess the effects of Chinese Baijiu with different flavors as supplementary material on microbial communities and flavor formation during inoculated fermentation of Chinese Dongbei Suancai. The results showed that the addition of Fen flavor Baijiu significantly increased the relative abundance of *Candida*, Luzhou flavor Baijiu increased the relative abundance of *Pedobacter* and *Hannaella*, while Maotai flavor Baijiu increased the *Chryseobacterium* and *Kazachstania*. A total of 226 volatile metabolites were detected in Suancai fermented when adding different flavors of Baijiu. Furthermore, the significantly upregulated metabolites (*p* < 0.01) of Suancai after adding Baijiu increased by 328.57%, whereas the significantly downregulated metabolites decreased by 74.60%. Simultaneously, the addition of Baijiu promoted the synthesis and decomposition of amino acids and short-chain fatty acids in the early and middle stages of fermentation. Further, Maotai flavor Baijiu improved the diversification of metabolic pathways in the late stage of Suancai fermentation. The E-nose response showed that sulfur-organic, broad-alcohol, sulfur-chlor was the principal differential flavor in Suancai caused by adding Baijiu with different flavors. Simultaneously, Fen flavor Baijiu and Luzhou flavor Baijiu accelerated the formation of the Suancai flavor. These results indicated that Baijiu with different flavors had significant effects on the flavor formation of inoculated fermented Suancai.

## 1. Introduction

Traditional Chinese Dongbei Suancai is a fermented salted vegetable with a salt concentration of 2–10%. It is prepared from Chinese cabbage and produces special flavors and nutrients through the metabolism of microorganisms in the brine [1]. In recent years, to accelerate the fermentation process and enhance food safety, the use of starters in Dongbei Suancai fermentation grew to be the subject of extensive study and became more commonplace in industrial production [2].

Flavor determines the acceptance of Chinese Dongbei Suancai among consumers. Research reports that the volatile flavor components of traditional naturally fermented Chinese Dongbei Suancai include alcohols, esters, alkanes, ketones, aldehydes, olefins, etc., [3]. Although more acids were found in the inoculated fermented Suancai, fewer varieties of volatile flavor compounds were detected when compared to naturally fermented Suancai [4].

At present, to improve the flavor of fermented fruits and vegetables, Chinese Baijiu, with its unique flavor, is usually added before fermentation in industrial production and research trials [5]. Chinese Baijiu, which has a diverse array of compounds associated with flavor, is a fermented drink popular with consumers in China. In particular, alcohols and esters are major contributors to the intense, volatile aroma [6]. Our previous preliminary study found that Luzhou flavor Baijiu as supplementary material altered the bacterial structure and non-volatile metabolite composition of Suancai [7]. However, volatile metabolites directly related to flavor need to be further analyzed. Furthermore, tracing the metabolic pathways of differential volatile metabolites is essential to get a deeper insight into the generation of varied flavors of Suancai.

Because Baijiu is usually divided into different flavors according to the aroma characteristics [8], the unique attributes of Baijiu need to be considered in depth. The typical flavors of Chinese Baijiu include Fen flavor, Luzhou flavor, Maotai flavor, etc. The main aroma type of Fen flavor Baijiu is grain flavor. Luzhou flavor Baijiu has a fruit flavor, sweet flavor, floral flavor and cellar-like flavor, which comes from esters [8]. The main flavor components of Maotai flavor Baijiu are sulfur-containing compounds that lead to a prominent aroma [9]. Therefore, different types of Baijiu added to Suancai will lead to differences in flavor and affect the stability of the quality of Suancai. Daqu is an important saccharification agent for the fermentation of Baijiu. It promotes the production of microorganisms and enzymes, which are essential for the flavor formation of Baijiu. However, the composition of microorganisms, especially fungi, in Daqu used to ferment Baijiu with different flavors varies, and it can cause discrepancies in the microflora and flavor of Suancai when various flavors of Baijiu are added [10]. However, few researchers have examined the effects of various flavors of Baijiu on the succession of fungal communities during Suancai fermentation and the effects of different Baijiu flavors on the Suancai flavor.

Therefore, in this study, the Fen flavor Baijiu, Luzhou flavor Baijiu, and Maotai flavor Baijiu were added as supplementary material to ferment Suancai, and the diversity of bacteria and fungi during Suancai fermentation was comprehensively analyzed. Analysis was based on the differential volatile flavor components, focusing on the differences in metabolic pathways and simultaneously identifying the different flavor profiles of Suancai by E-nose. The purpose was to provide a comprehensive and in-depth evaluation of the discrepancies in bacterial and fungal diversity, volatile flavor components, and metabolic pathways of inoculated fermented Suancai when adding different Baijiu and analyzing the effects of various Baijiu on the flavor structure of Suancai. The results contribute to the understanding of the flavor formation mechanism of Suancai with different types of Chinese Baijiu as supplementary material. Simultaneously, this study provides theoretical guidance for its industrial production to improve the flavor quality of Chinese Dongbei Suancai.

## 2. Materials and Methods

### 2.1. Preparation of Mixed Bacterial Starter Culture

In this study, *Lactiplantibacillus plantarum* (*L. plantarum*) and *Leuconostoc citreum* (*L. citreum*), with excellent fermentation properties were used. *L. plantarum* LNJ002 (CGMCC No. 20610) was isolated from Chinese Dongbei Suancai produced at low temperatures in our laboratory, and *L. citreum* BNCC 194779 was obtained from BeiNa Chuanglian Biotechnology Co., Ltd. (Suzhou, China) in November 2020. The two lactic acid bacteria (LAB) strains were cultured anaerobically in the MRS medium at 30 °C for 24 h according to the method of Li et al. [1]. Subsequently, sterile physiological saline was used to respectively adjust the bacterial cell density to approximately 10^9^ CFU/mL. A mixed starter culture of *L. plantarum* and *L. citreum* (1:1) was prepared.

### 2.2. Preparation of Suancai Samples

Fresh Chinese cabbages were obtained from the Nongkeyuan market in Shenyang, Liaoning Province, China. The inoculated fermented Dongbei Suancai was prepared according to the method described by Li et al. [1] with slight modification. For preparation before sealing, the Fen flavor Baijiu (60 ± 1 vol%), Luzhou flavor Baijiu (60 ± 1 vol%) and Maotai flavor Baijiu (53 ± 1 vol%) with a concentration of 2% (*v*/*w*) were added, respectively. The different flavor types of Chinese Baijiu were purchased from Shenyang Nuolian Technology Co., Ltd., Shenyang, China. The sealed plastic jars were placed at 22 ± 1 °C for 21 days. There were four different batches made: a control (only starter culture added, control, C), adding Fen flavor Baijiu (starter culture and Fen flavor Baijiu added, Fen group, F), adding Luzhou flavor Baijiu (starter culture and Luzhou flavor Baijiu added, Luzhou group, L), and adding Maotai flavor Baijiu (starter culture and Maotai flavor Baijiu added, Maotai group, M). Equal amounts of Suancai brine were collected respectively from the upper, middle, and under layers of each plastic jar. Simultaneously, the samples were collected from four different Suancai batches on days 1, 8, and 22 for bacterial and fungal diversity analysis and metabolite analysis. ‘C0’, ‘C7’, ‘C21’, ‘F0’, ‘F7’, ‘F21’, ‘L0’, ‘L7’, ‘L21’, and ‘M0’, ‘M7’, and ‘M21’ were used to represent the samples of four batches at the three times, respectively. The analysis of bacterial and fungal diversity referred to Li et al.; the separated pellets were collected after centrifugation at 10,000× *g* for 10 min [1]. The samples for metabolite analysis were centrifuged at 2000× *g* for 20 min, and the supernatants were collected.

### 2.3. DNA Extraction and Illumina MiSeq Sequencing

The DNA extraction and polymerase chain reaction amplification were conducted according to Li et al. [1]. A NanoDrop 2000 UV-vis spectrophotometer (Thermo Fisher Scientific, Wilmington, NC, USA) was used to determine the DNA concentration and purity. An ABI GeneAmp^®^ 9700 PCR thermocycler (ABI, Loma Linda, CA, USA) was used to conduct polymerase chain reaction (PCR) amplification. Primer pairs 338F (5′-ACTCCTACGGGAGGCAGCAG-3′) and 806R (5′-GGACTACHVGGGTWTCTAAT-3′) were used to amplify the hypervariable region V3–V4 of the bacterial 16S rRNA gene. Primer pairs ITS1F (5′-CTTGGTCATTTAGAGGAAGTAA-3′) and ITS2R (5′-GCTGCGTTCTTCATCGATGC-3′) were used to amplify the fungal ITS sequence region of fungal rRNA gene. An Illumina MiSeq PE300 platform (Illumina, San Diego, CA, USA) was used to purify and pool the bacterial and fungal PCR products. The fastp (Version 0.20.0) was used to quality-filter the raw FASTQ files from MiSeq sequencing. The SILVA database (version 138.1/16s_bacteria) and the Unite database (version 8.0/its_fungi) were used to analyze the taxonomy of each bacterial and fungal sequence in a confidence threshold of 70%.

### 2.4. Volatile Metabolites Profiling by GC-MS

The volatile metabolites were determined according to the method described by Ding et al. [11] with some modifications. The 100 μL Suancai brine was mixed with 300 μL of methanol: acetonitrile (*v*/*v* = 2:1) containing internal standard (0.02 mg/mL L-2-chlorophenylalanine). The sample was cryogenic sonicated for 30 min, then centrifuged at 13,000× *g* for 15 min at 4 °C. Subsequently, 80 μL of methoxypyridine hydrochloride solution (15 mg/mL) was added and shaken at 35 °C for 95 min in a shaken incubator. Then 80 μL BSTFA (containing 1% TMCS) derivatization reagent was added, and the reaction was allowed to progress to 70 °C for 65 min. The samples were removed and left at 25 °C for 30 min for gas chromatography-mass spectrometry (GC-MS) analysis.

The GC-MS analysis was performed on an Agilent 8890B gas chromatography coupled with an Agilent 5977B mass selective detector (Agilent, Santa Clara, CA, USA). The instrument was equipped with an inert electron impact (EI) ionization source with an ionization voltage of 70 eV (Agilent, USA). A DB-5MS (40 m × 0.25 mm × 0.25 µm) capillary column was used to separate the samples. The carrier gas was helium at a flow rate of 1 mL/min. The GC column temperature was kept at 60 °C for 30 s and rose to 310 °C at a rate of 8 °C/min, kept constant at 310 °C for 6 min. The 1 µL sample was injected at an inlet temperature of 300 °C, and the split ratio was 10:1. The mass spectrometry conditions were as follows: ion source temperature, 280 °C; quadrupole temperature, 150 °C; quality scanning range, 50–500 m/z; the scanning frequency, 3.2 scan/s.

### 2.5. E-Nose Analysis

The E-nose analysis was performed according to the description by Chen et al. with modifications [12]. A commercial PEN 3.5 E-nose (Airsense Analytics, Schwerin, Germany) containing 10 metal-oxide gas sensors was used for the headspace analysis. The sensor flush time was 80 s, zero-point trim time was 10 s, and both chamber and initial injection flow were 300 mL/min. The sample interval was 1 s, and the sample measurement time was 120 s. Each sample was assayed three times.

### 2.6. Statistical Analysis

The data were analyzed on an online platform of Majorbio Cloud Platform (www.majorbio.com). The R package “ropls” (Version 1.6.2) was used to perform principal component analysis (PCA). The *p* < 0.01 and |Log2fold change (FC)| > 1 were chosen as the threshold for the analysis of significant metabolite expression differences.

## 3. Results and Discussion

### 3.1. Microbial Communities during Suancai Fermentation When Various Flavors of Baijiu Were Added

#### 3.1.1. Microbial Diversity in Suancai with Addition of Various Flavors of Baijiu

After trimming the raw readings and deleting mitochondria and chloroplasts, RNA gene sequencing yielded a total of 288 fungal and 144 bacterial OTUs (Operational Taxonomic Units). The numbers of bacterial OTUs and fungi OTUs in control, and the three groups of Suancai samples with the addition of Baijiu were decreased during fermentation. Simultaneously, four sets of Suancai sample groups exhibited a declining tendency in the fermentation process, as shown by the bacterial and fungal Shannon, Ace, and Chao indices. Due to the inoculation, the acidity of the fermentation environment increased rapidly, and it prevented the development of bacteria and fungi with low acid tolerance; the microbial communities’ richness and diversity gradually decreased in the process of inoculated fermentation with or without the addition of Baijiu [4]. In particular, the OTU number and alpha diversity indices dropped sharply in the first 8 days of fermentation. Referring to previous studies, the results of day one of fermentation represented the average indicator of the early fermentation stage, the results of day 8 represented the average indicator of the middle fermentation stage, the results of day 22 represented the average indicator of the late fermentation stage based on the changes in pH during the Suancai fermentation [7]. Therefore, the microbial community structures changed significantly in the early and middle stages of Suancai fermentation. Besides, on day 22 of fermentation, the fungal community of Suancai added with Maotai flavor Baijiu showed the smallest Shannon index (0.24 ± 0.13), Ace index (54.23 ± 17.03) and Chao index (52.67 ± 19.11). It was proposed that the diversity and richness of fungal communities were significantly impacted by the Maotai flavor Baijiu.

#### 3.1.2. Microbial Community Structures of Suancai with Addition of Various Flavors of Baijiu

**Figure 1:**
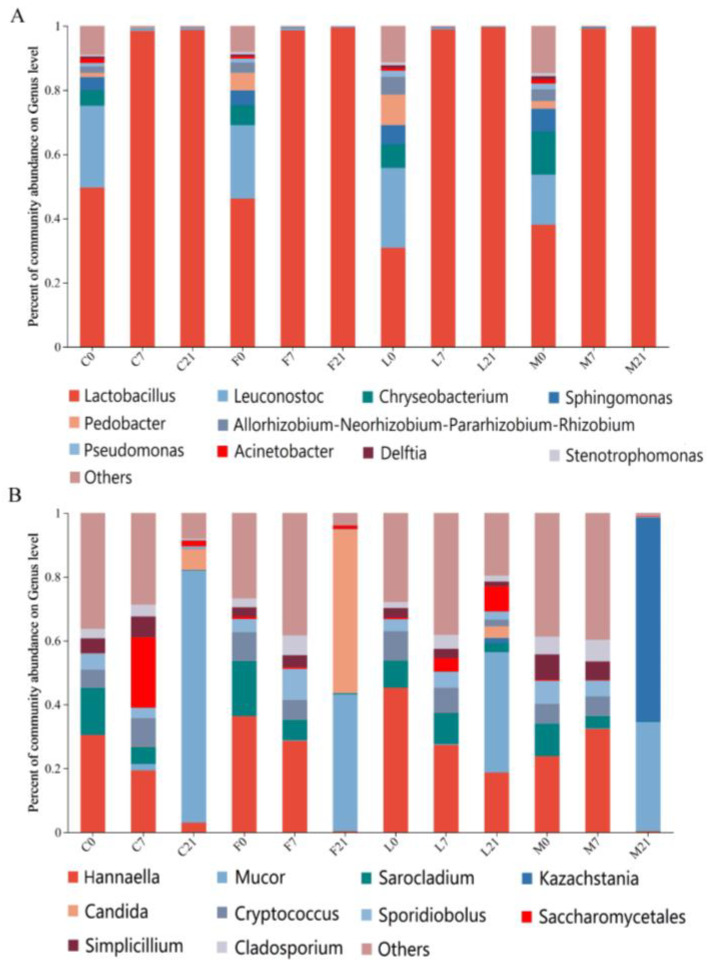
Species composition analysis. (**A**) Bacterial community composition at the genus level; (**B**) Fungal community composition at the genus level.

Classification at the genus level was used to examine the bacterial and fungal community architectures during Suancai fermentation (Figure 1A,B). For bacteria, due to the exogenous LAB being added, the relative abundances of *Lactobacillus* (30.82–49.64%) and *Leuconostoc* (15.60–25.46%) in Suancai with and without Baijiu were high on day 1 of fermentation, followed by *Chryseobacterium*, *Sphingomonas*, *Pedobacter*, *Rhizobium*, *Pseudomonas*, *Acinetobacter*, *Delftia*, and *Stenotrophomonas* (Figure 1A). However, Song et al. [13] found that the dominant bacteria in the early stage of spontaneous fermentation of Suancai included *Serratia*, *Rahnella*, *Shewanella*, etc. The diversity of bacteria in Suancai was different due to environmental factors and fermentation mode. The predominant bacterial genera found in fresh-cut vegetables were *Pseudomonas*, *Acinetobacter* and others [14], and their relative abundance gradually decreased during fermentation. Besides, *Leuconostoc* (as heterofermentative LAB) was active in the early fermentation stage. However, because of their low acid tolerance, the acidic environment hindered their development, and the relative abundance thereof decreased in the middle and late fermentation stages [15]. On the contrary, along with the fermentation, *Lactobacillus* becomes the dominant genus, which was similar to the findings of previous research [16]. Different from naturally fermented Suancai, due to the exogenous *Lactobacillus* and *Leuconostoc* had negative interactions with *Enterobacter* and *Pediococcus*, the amount of *Enterobacter* and *Pediococcus* was low in the inoculated Suancai [17]. Furthermore, the relative abundance of *Pedobacter* increased, and the relative abundance of *Lactobacillus* dramatically reduced with the addition of Luzhou flavor Baijiu, while the relative abundance of *Chryseobacterium* and *Sphingomonas* increased and the relative abundance of *Leuconostoc* decreased significantly with the addition of Maotai flavor Baijiu. Due to the introduction of complex LAB by Luzhou and Maotai flavor Baijiu, the LAB community structures in the original Suancai were changed.

The fungal diversity had significant differences during Suancai fermentation (Figure 1B). *Hannaella* (23.81–45.29%), *Sarocladium* (8.54–17.29%), *Cryptococcus* (5.76–9.05%), *Sporidiobolus* (3.78–7.20%), *Saccharomycetales* (0.06–0.8%), *Simplicillium* (2.89–7.91%) and *Cladosporium* (1.90–5.58%) were founded in all samples on the first day of fermentation. However, adding Baijiu significantly changed the fungal abundance of inoculated Suancai. Fen flavor Baijiu increased the *Cryptococcus*, *Sarocladium*, *Saccharomycetales*; Luzhou flavor Baijiu increased the *Hannaella*, *Cryptococcus*, *Saccharomycetales*, and Maotai flavor Baijiu increased the *Sporidiobolus*, *Saccharomycetales*, *Simplicium*, and *Cladosporium*. It was worth noting that on day 8 of fermentation, *Saccharomycetales* accumulated rapidly in the inoculated Suancai without Baijiu. Because the *Saccharomycetales* existed in Baijiu, they appeared at the beginning of Suancai fermentation when Baijiu was introduced into Suancai [18]. In addition, during Suancai fermentation, the *Hannaella*, *Sarocladium*, *Sporidiobolus* and *Simplicium* showed decreasing trends in the control and three Baijiu groups. *Mucor* appeared and grew rapidly in all four groups of samples in the middle and late fermentation stages. However, *Kazachstania* only appeared and grew rapidly in the Maotai group. Therefore, *Mucor* was the dominant genus for Suancai fermentation; further, *Kazachstania,* introduced by the Maotai flavor Baijiu, also became the dominant genus [19].

#### 3.1.3. Differences between Microbial Communities of Suancai Fermented with Various Flavors of Baijiu

**Figure 2:**
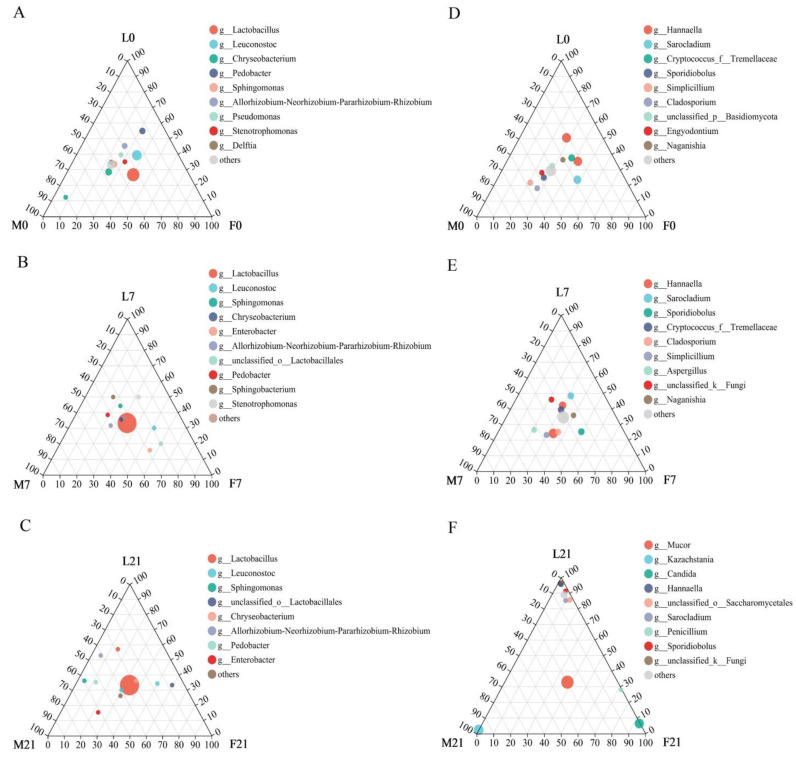
The ternary phase diagram of bacterial and fungal succession in Suancai fermented with adding Baijiu. (**A**) The bacteria on day 1 of Suancai fermentation; (**B**) The bacteria on day 8 of Suancai fermentation; (**C**) The bacteria on day 22 of Suancai fermentation; (**D**) The fungi on day 1 of Suancai fermentation; (**E**) The fungi on day 8 of Suancai fermentation; (**F**) The fungi on day 22 of Suancai fermentation.

The composition and distribution of the dominant genus in samples of three Baijiu groups during fermentation were shown by the ternary phase diagram (Figure 2). On day 1 of fermentation, exogenically added *Lactobacillus* was dominant in three groups of Baijiu samples, and its dominant effect in three groups of Baijiu samples became increasingly obvious during fermentation. Wang et al. [20] believed that the *Leuconostoc* disappeared in the late stage of Paocai fermentation. In this study, exogenically added *Leuconostoc* exhibited high activity in the early fermentation stage; subsequently, it rapidly decreased in the middle and late fermentation stages; however, it could maintain the low abundance in the samples of the Fen group and Luzhou group. It also suggested that although the acid tolerance of *Leuconostoc* was poor, the Maotai flavor Baijiu had a stronger inhibitory influence on the growth of *Leuconostoc* than the other two flavors of Baijiu. In addition, *Pedobacter* and *Sphingomonas* exhibited high abundance in the samples of three Baijiu groups at the beginning of fermentation, subsequently decreased with fermentation, and retained their original low abundance only in the samples of Maotai and Luzhou groups in the late fermentation stage. It is worth noting that *Enterobacter,* which had a high abundance in naturally fermented Suancai, appeared at the middle fermentation stage in inoculated fermented Suancai when added Baijiu. Further, it retained a higher abundance in the Maotai group than in the Fen and Luzhou groups in the late fermentation stage. Therefore, Suancai samples of the Maotai group could retain the greater richness of LAB in the late fermentation stage, which was consistent with the aforementioned results of the LAB diversity assay. For fungi, in early and middle fermentation stages, different fungi in the Suancai samples of three Baijiu groups have high abundance and typical distribution. However, in the late fermentation stage, the dominant genus of the three groups of samples had significantly different distributions. *Sporidiobolus* existed in all three groups; *Kazachstania* and *Candida* maintain high abundance in the Maotai group and Fen group, while more fungi only retain their activity in the Luzhou group.

**Figure 3:**
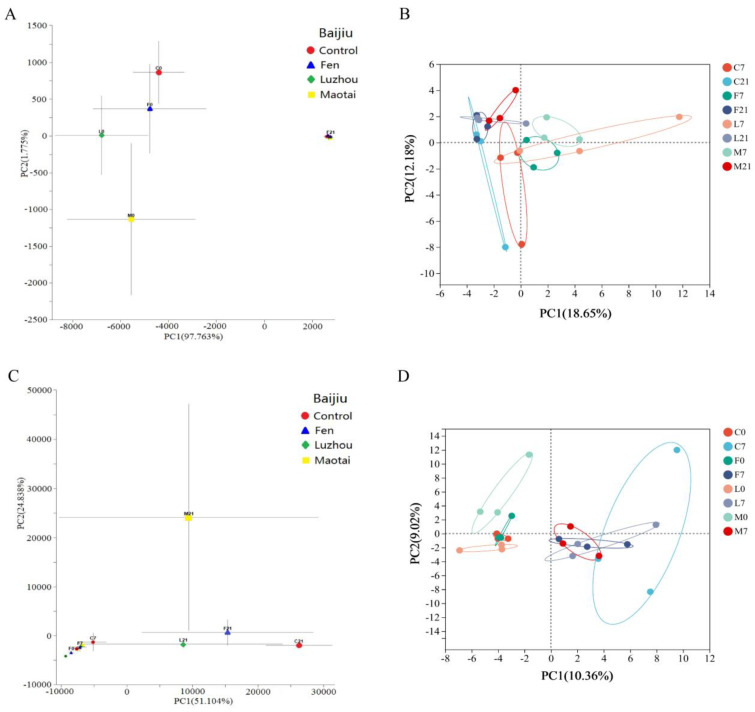
Difference analysis in the bacterial and fungal community structure of Suancai with added Baijiu. (**A**) Multiple principal component analysis (MPCA) of bacteria; (**B**) Principal component analysis (PCA) of bacteria from day 8 to 22 of Suancai fermentation; (**C**) MPCA of fungi; (**D**) PCA of fungi from day 1 to 8 of Suancai fermentation.

The microbial communities of the Control and Baijiu groups at different stages of fermentation were analyzed by multiple principal component analysis (MPCA). For bacteria, it was found that there were significant differences in sample groups between day 1 of fermentation and other fermentation times. Simultaneously, significant differences also existed between the Control and Baijiu groups on day 1 of fermentation (Figure 3A). For fungi, significant differences arose between the sample groups on day 22 of fermentation and other groups (Figure 3C). To assess the distinctions in bacterial and fungal community structures of Suancai fermented with various flavors of Baijiu, principal component analysis (PCA) was used to analyze the microbial communities of Control and Baijiu groups on days 8 and 22 of fermentation for bacteria and days 1 and 8 of fermentation for fungi. The bacterial community structures on days 8 and 22 of fermentation were clustered into separate groups, respectively. Simultaneously, the fungal community structures on days 1 and 8 of fermentation were clustered into separate groups, respectively (Figure 3B,D). However, the microbial community structures of the control and different Baijiu groups on the same day of fermentation were relatively discrete, suggesting that the addition of Baijiu significantly changed the bacterial and fungal community structures in Suancai, and fermentation time had a greater influence on the microbial community structures in Suancai. Since certain LAB’s poor resistance to the fermentation environment, the diversity of bacteria was decreased, and the bacterial structures of Suancai became comparable and stable until the late fermentation stage. Therefore, the dispersion of bacterial community structure gradually decreased during fermentation, regardless of the addition (or not) of Baijiu. For fungi, due to the abundance of fungi in Baijiu, the different fungi introduced by different flavors of Baijiu at the beginning of fermentation began to grow as fermentation progressed, leading to significant differences in fungal community structure at middle and late fermentation stages [10].

### 3.2. Volatile Metabolite Profiles in Suancai Fermentation with Addition of Various Flavors of Baijiu

**Figure 4:**
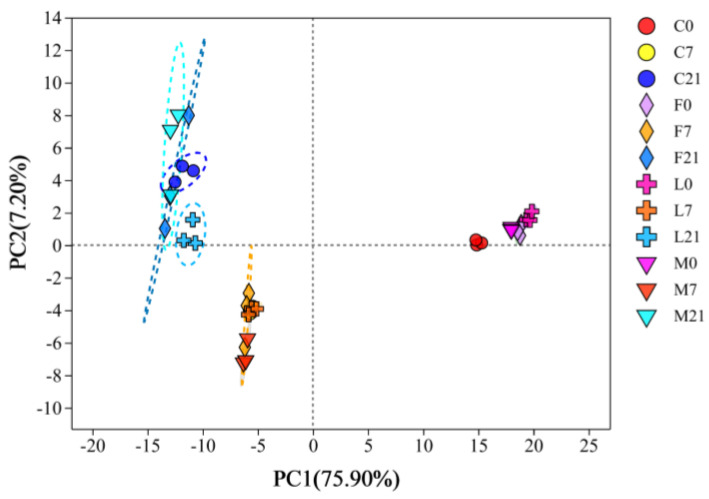
The PCA analysis of volatile metabolites of Suancai fermented with the addition of Baijiu.

In all Suancai sample groups, 226 volatile metabolites in total were found. The dominant one was organic acids and derivatives (41.30%), followed by organic oxygen compounds (17.39%), organoheterocyclic compounds (13.04%), benzenoids (7.61%), lipids and lipid-like molecules (7.61%), organic nitrogen compounds (6.52%), and other compounds. Figure 4 displays the PCA analysis of the volatile metabolites in Suancai. The variance contribution rates of the first two principal components were 75.90% and 7.20%. The volatile metabolites of Suancai on days 1, 8 and 22 of fermentation were grouped as a distinct group, respectively. It suggested that at different fermentation periods, Suancai’s volatile metabolites differed noticeably. Simultaneously, the difference in principal coordinate (PC) showed that the volatile metabolites on day 8 and day 22 of fermentation were closer, which suggested that the volatile metabolites of Suancai exhibited greater differences in the early fermentation stage. Furthermore, at the beginning of fermentation, the samples of the Baijiu groups and the samples of the control were clustered into one group, respectively, whereas the dispersion of volatile metabolites of the samples in different Baijiu groups gradually increased as fermentation progressed. The various Baijiu flavors led to the significant difference in Suancai volatile metabolites in the late fermentation stage. Due to the introduction of Baijiu compounds, the differences between the Control and Baijiu groups were significant at the beginning of fermentation. On the other hand, different microorganisms introduced by Baijiu metabolized new volatile metabolites in the middle and late fermentation stages, leading to the differences in the Baijiu groups with different flavors in the late fermentation stage [7].

### 3.3. Metabolic Differences of Suancai with Addition of Various Flavors of Baijiu

**Figure 5:**
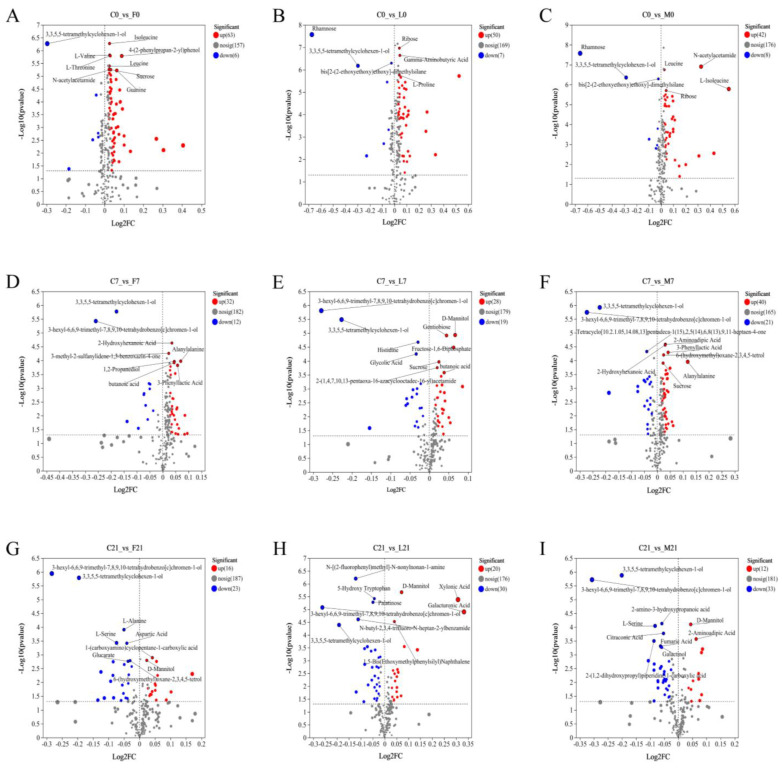
Differential metabolite analysis of Suancai fermented with addition of Baijiu. (**A**) Control on day 1 v. Fen group on day 1; (**B**) Control on day 1 v. Luzhou group on day 1; (**C**) Control on day 1 v. Maotai group on day 1; (**D**) Control on day 8 v. Fen group on day 8; (**E**) Control on day 8 v. Luzhou group on day 8; (**F**) Control on day 8 v. Maotai group on day 8; (**G**) Control on day 22 v. Fen group on day 22; (**H**) Control on day 22 v. Luzhou group on day 22; (**I**) Control on day 22 v. Maotai group on day 22.

The differentially expressed metabolites in Suancai are shown in Figure 5, and *p* < 0.05 was used as the cut-off threshold of significant difference in the volcano plot. Compared to the control group, the Fen group included 6 significantly upregulated metabolites and 63 significantly downregulated metabolites on day 1 of fermentation, the Luzhou group included 7 significantly upregulated metabolites and 50 significantly downregulated metabolites, the Maotai group included 8 significantly upregulated metabolites and 42 significantly downregulated metabolites. Subsequently, the significantly upregulated metabolites in the three Baijiu groups increased gradually, whereas the significantly downregulated metabolites decreased as fermentation progressed. It suggested that adding different flavors of Baijiu changed the volatile metabolite composition during Suancai fermentation. Besides, on day 1 of fermentation, due to more volatile metabolites being introduced into Suancai by Maotai flavor Baijiu, the Maotai group had the most significantly upregulated metabolites. Furthermore, most of the differential volatile metabolites, including amino acids, organic acids acetamide, etc., simultaneously appeared in at least two Baijiu groups, especially 3,3,5,5-tetramethylcyclohexen-1-ol belonged to the common differential metabolites of three Baijiu groups, while rhamnose was the specific differential metabolite in specimens in the Luzhou group. Simultaneously, malic acid and piperidine-2-carboxylic acid were the specific significantly downregulated metabolites of Fen group, 4-hydroxybutanoic acid and 8-aminoquinolin-6-ol were the specific significantly downregulated metabolites of Luzhou group, and L-isoleucine, succinic acid, erythritol and 1-[(2-methylpropan-2-yl)oxy]propan-2-ol were the specific significantly downregulated metabolites of Maotai group. Therefore, the addition of Baijiu accelerated the metabolism of organic acids and amino acids during the early fermentation of Suancai. It was noteworthy that Chinese Baijiu was rich in esters and alcohols [6]; however, it was not found in the top 10 differential metabolites. It might be due to less Baijiu having been added to the Suancai and the alcohols and esters involved in the metabolic transformation during Suancai fermentation. Hence, the difference in esters and alcohols was low. In addition, the Luzhou group and Maotai group simultaneously had more upregulated metabolites and downregulated metabolites in the middle and late fermentation stages, indicating that Luzhou flavor Baijiu and Maotai flavor Baijiu had significant effects on the transformation in Suancai metabolites. Further, adding various flavors of Baijiu resulted in the production of more amino acids, organic acids, nitrogenous compounds and heterocyclic compounds in late Suancai fermentation, which were significantly related to the taste and flavor of Chinese Dongbei Suancai. Therefore, Baijiu could change the flavor component structure in Suancai, thereby affecting the sensory quality of Suancai.

**Figure 6:**
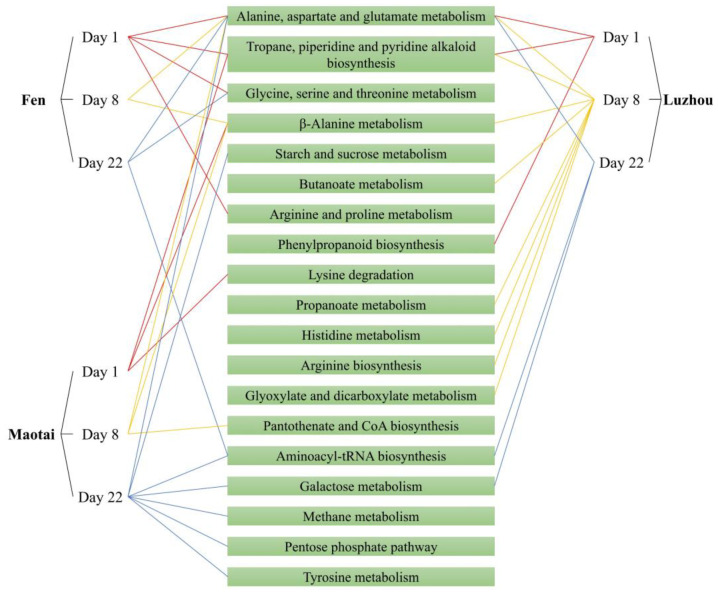
Differential metabolic pathways in Suancai with addition of Baijiu with different flavors obtained by differential metabolite enrichment.

The differential metabolites in the fermentation process of Suancai with the addition of three flavors of Baijiu were enriched, and the hypergeometric distribution algorithm was used to obtain the pathway of significant enrichment of differential metabolites, as shown in Figure 6. In the Suancai samples of three Baijiu groups, a variety of differential metabolites were significantly enriched and showed importance in the pathways of amino acid metabolism, aminoacyl-tRNA biosynthesis, carbohydrate metabolism, short-chain fatty acid metabolism, etc. In the early and middle fermentation stages, the differential metabolic pathway of three Baijiu group samples focused on amino acid metabolism simultaneously. Suancai added with Fen flavor Baijiu and Luzhou flavor Baijiu also involved short-chain fatty acid metabolism and glyoxylate and dicarboxylate metabolism. However, the differential metabolic pathways of Suancai treated with three flavors of Baijiu were diversified during late fermentation; specifically, the differential metabolic pathways in Maotai group samples of Suancai were more complex. The differential metabolic pathway gradually shifted from amino acid metabolism to aminoacyl-tRNA biosynthesis, carbohydrate metabolism, methane metabolism, pentose phosphate pathway, etc. It indicated that Baijiu promoted the metabolism of short-chain fatty acids and amino acids during early and middle fermentation. However, the influence of different flavors of Baijiu on the amino acid metabolism in the late stage of fermentation was decreased. Furthermore, the Maotai flavor Baijiu promoted the diversification of metabolic pathways during late Suancai fermentation. Consequently, it was believed that the Maotai flavor of Baijiu had a greater impact on the volatile metabolites of Suancai.

### 3.4. Correlation Analysis between Volatile Metabolites and Microbiota in Suancai with Addition of Various Flavors of Baijiu

**Figure 7:**
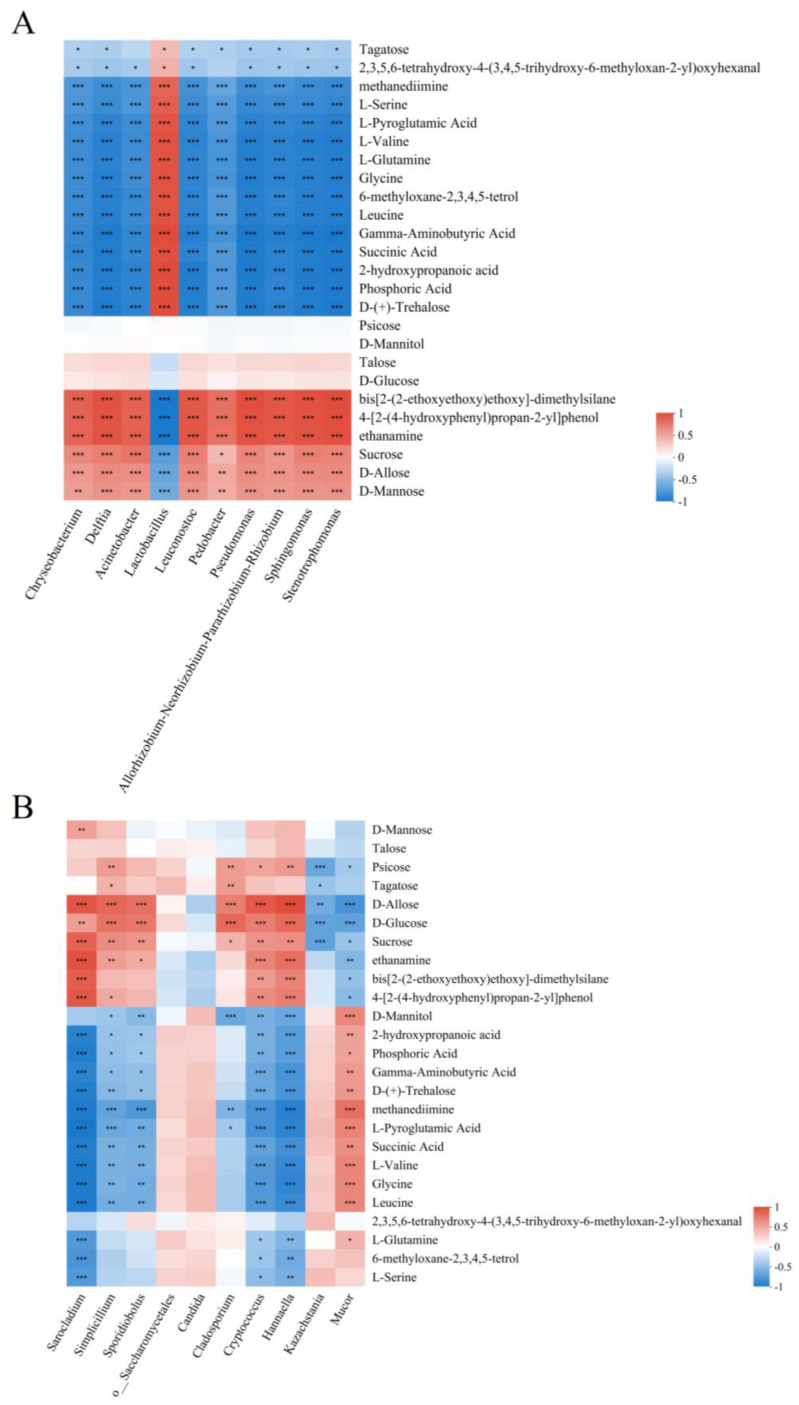
Correlation between microbiota and cell wall components during Suancai fermentation. The red color represented the positive correlation, and the blue color represented the negative correlation. “*” represented 0.01 < *p* ≤ 0.05, “**” represented 0.001 < *p* ≤ 0.01, “***” represented *p* < 0.001. (**A**) Correlation between bacterial genera and volatile metabolites of Suancai with adding Baijiu; (**B**) Correlation between fungal genera and volatile metabolites of Suancai with adding Baijiu.

The top 25 volatile metabolites and the top 10 microbiotas of Suancai with various flavors of Baijiu added were used to draw the correlation heat-map diagrams. *Lactobacillus* was positively correlated with alkanes, organic acids, short-chain fatty acids and amino acids, whereas other LABs showed positive correlations with dimethylsilane, ethanamine and saccharide (Figure 7A). The result was similar to the one found by Liang et al. [21], that *Lactobacillus* was significantly positively correlated with acids, hydrocarbons and heterocycles. Simultaneously, *Leuconostoc* was found to positively correlate with organic acid in other studies [4]. Nonetheless, the current investigation revealed some alterations in the correlation between *Leuconostoc* and metabolites. As heterofermentative LAB, *Leuconostoc* could contribute to enriching the flavor of Suancai. At present, *Leuconostoc mesenteroides* (*L. mesenteroides*) is mainly isolated from Suancai. Simultaneously, *L. mesenteroides* was used in other studies to ferment Suancai. However, to produce different flavors of Suancai, *L. citreum* was inoculated in the present study, which might lead to different correlations between *Leuconostoc* and volatile metabolites. On the other hand, because the components of Baijiu act as precursor substances in Suancai’s metabolism, the composition of volatile metabolites in Suancai was changed. Hence, the differences in the correlation between *Leuconostoc* and volatile metabolites emerged [7]. For fungi, a positive correlation was displayed between amino acids and *Mucor* (Figure 7B). It indicated that *Mucor* had an important influence on the flavor formation of Suancai. Besides, *Sarocladium*, *Hannaella*, *Cryptococcus*, *Sporidiobolus* and *Simplicillium* had positive correlations with saccharides. Science cellulose and pectin were crucial components that composed the cell wall, and they were essential for providing the textural characteristics of fruits and vegetables [22]. Further, pectin and cellulose decomposed to produce neutral sugars under the catalysis of pectinase and cellulase [23]. Therefore, these fungi were considered to produce pectinase and cellulase during Suancai fermentation, causing the decomposition of pectin or cellulose and the generation of neutral sugars. Ren et al. [24] also believed that fungi play an important role in softening the texture of fruit and vegetable products.

### 3.5. E-Nose Response to Suancai Samples

**Figure 8:**
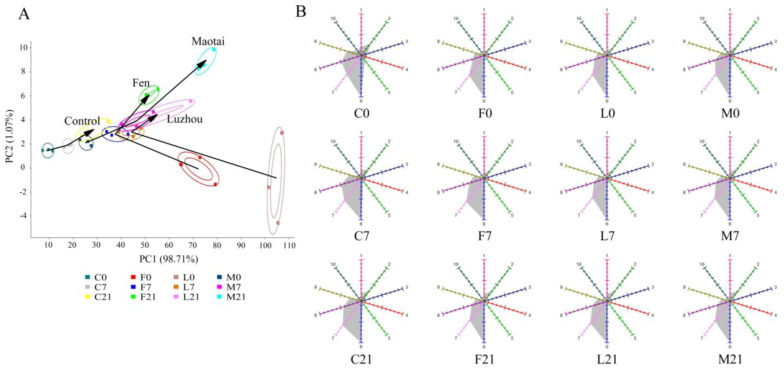
E-nose responses of different Suancai samples. (**A**) PCA of flavor characteristics of the Control and three Baijiu groups; (**B**) Responses of different Suancai samples to sensors.

E-nose was considered a method of visualizing (and differentiating) changes in flavors. Therefore, it was used to analyze the flavor differences between the control and Baijiu groups of Suancai samples during fermentation. The responses of different Suancai samples to sensors (1–10) of the E-nose are represented in Figure 8. Sensors 1–10 represented aromatic, broad range, sensitivity to aromatic components, hydrogen, arom-aliph, broad-methane, sulfur-organic, broad-alcohol, sulfur-chlor, and methane-aliph, respectively [12]. Sensors 2, 6, 7, 8, and 9 had high response values in each group, indicating that broad range, broad-methane, sulfur-organic, broad-alcohol, and sulfur-chlor were the dominant flavors of inoculated fermented Suancai. There were no significant differences in sensors 6 and 7 in each group on day 1 of fermentation. However, the control had a stronger response from sensors 2, 8 and 9 than when used to assay Baijiu groups. Due to the samples of Baijiu groups contained more methane and terpenes (or structure-like) and sulfur-containing organic compounds such as bis[2-(2-ethoxyethoxy)-ethoxy]-dimethylsilane (significantly upregulated metabolite in Baijiu group samples), etc., the response of sensors 2, 8, and 9 were weakened. In the late stage of fermentation, compared with the control, Fen group samples had a lower response at sensor 8, Luzhou group samples had a lower response at sensor 9, and Maotai group samples had lower responses at both sensors 7 and 8. It indicated that the addition of Baijiu affected the flavor structure of Suancai, and different flavors of Baijiu led to the different flavor structures in Suancai. Simultaneously, structural differences were mainly reflected in the three flavors of sulfur-organic, broad-alcohol, and sulfur-chlor.

Visualization of flavor characteristics of Suancai samples of the control and three Baijiu groups is shown in Figure 8A. A total variance of 99.78% was explained by the first two principal components; hence, the first two PCs were sufficient to explain the total variance of the dataset. Four groups of Suancai samples were distinguished from each other on day 1 of fermentation, which suggested that Suancai added with different flavors of Baijiu had significantly different flavors in the early fermentation stage. It was worth noting that the Suancai samples of the Fen group were similar on day 8 and day 22 during the fermentation process, and a similar phenomenon arose in the Luzhou group, indicating that the unique flavor of Suancai with Fen flavor Baijiu and Luzhou flavor Baijiu added was formed in the middle fermentation stage. Simultaneously, from the perspective of the changes in the directions of flavor difference, the flavor metabolism pathways of the Fen and Luzhou groups were similar. Furthermore, because the addition of the Maotai flavor Baijiu had significant effects on the flavor of Suancai, the Suancai fermented with the added Maotai flavor Baijiu was far away from the control in the late fermentation stage.

## 4. Conclusions

The microbial community, metabolic pathways and flavor characteristic response during fermentation of Suancai with various flavors of Baijiu added were assessed in this study. The addition of Baijiu significantly affected the bacterial community structures during early Suancai fermentation and the fungal community structures during late fermentation. Further, Luzhou flavor Baijiu and Maotai flavor Baijiu inhibited the growth of Lactobacillus and Leuconostoc during early Suancai fermentation, respectively. Fen flavor Baijiu and Maotai flavor Baijiu promoted the growth of Candida and Kazachstania during late Suancai fermentation, respectively. In addition, Luzhou and Maotai flavor Baijiu exerted more significant influences on the volatile metabolites in the middle and late fermentation stages, generating more amino acids, organic acids, nitrogenous compounds and heterocyclic compounds. Amino acid metabolism was the most important differential metabolic pathway in the early and middle fermentation of the Suancai samples of the Baijiu groups. Moreover, due to the differential metabolic pathways of the Maotai group in the late fermentation stage being more diverse, the Maotai flavor Baijiu had more significant effects on the volatile metabolites of Suancai. Due to LAB being significantly positively correlated with more flavor components, LAB was the dominant bacteria affecting the flavor formation of Suancai with Baijiu added. Furthermore, the Baijiu significantly affected the flavor in the early fermentation of Suancai. Subsequently, the flavor of Suancai with Fen and Luzhou flavor Baijiu added were almost formed in the middle fermentation stage, while Maotai flavor Baijiu still affected the flavor in the late fermentation stage of Suancai. The results provide a better understanding of the flavor characteristics of Suancai with Chinese Baijiu added. Simultaneously, the study results can guide in enhancing the flavor of inoculated fermented Suancai.

## Data Availability

The original contributions presented in the study are included in the article, further inquiries can be directed to the corresponding author.
